# Severe sepsis induced by zoledronic acid: A case report

**DOI:** 10.1097/MD.0000000000039188

**Published:** 2024-08-30

**Authors:** Lankai Liao, Ziyu Zhang

**Affiliations:** a Department of Emergency Medicine, Chengdu Fifth People’s Hospital, Chengdu, China; b Department of Clinical Pharmacology, Chengdu Fifth People’s Hospital, Chengdu, China.

**Keywords:** adverse reactions, Behcet disease, case report, severe osteoporosis, severe sepsis, zoledronic acid

## Abstract

**Rationale:**

Zoledronic acid is one of the most commonly used intravenous, highly potent amino diphosphonate salts worldwide and is commonly used in patients with primary or secondary osteoporosis, most of whom are well tolerated. There are currently no reports of severe sepsis induced by zoledronic acid. Here we present the first case of severe sepsis induced by zoledronic acid. We reviewed the literature and found that there is currently a lack of reports on severe sepsis induced by zoledronic acid or other diphosphonates.

**Patient concerns:**

A 58-year-old woman with severe osteoporosis and Behcet disease developed severe sepsis after treatment with zoledronic acid.

**Diagnosis:**

Sepsis, septic shock, acute kidney injury, intestinal infection, Behcet disease.

**Interventions:**

The patient was given intensive care immediately after admission, and massive fluid infusion to expand blood volume could not maintain normal blood pressure. Metaraminol was added to maintain circulatory stability, piperacillin–tazobactam was used to strengthen anti-infection, and glucocorticoids were used for anti-inflammatory treatment.

**Outcomes:**

The patient was discharged with improvement and followed up in the outpatient clinic.

**Lessons:**

The inducing mechanism of zoledronic acid is not clear, but when the patient has immunosuppression, the use of zoledronic acid should be cautious and monitored. In conclusion, severe sepsis induced by zoledronic acid is a rare but serious complication, and physicians should be aware of this adverse event in time to avoid serious consequences.

## 1. Introduction

Zoledronic acid is one of the most commonly used intravenous, highly potent amino diphosphonate worldwide, often used in patients with primary or secondary osteoporosis. Zoledronic acid, a nitrogen-containing third generation diphosphonate, will be strongly localized to the bone surface and absorption is particularly high at sites of increased bone turnover.^[1]^ Osteoclasts absorb bisphosphonates from the bone matrix resorption cavity, and then bisphosphonates trigger osteoclasts to undergo apoptosis, thereby inhibiting the bone resorption of osteoclasts and improving osteoporosis.^[2]^ We reviewed the literature and found that common adverse effects of bisphosphonate therapy in clinical patients were nephrotoxicity, acute phase reactions, gastrointestinal toxicity, and osteonecrosis of the jaw. Reports of severe sepsis induced by zoledronic acid are lacking. Therefore, we present the details of a case of severe sepsis induced by zoledronic acid.

## 2. Case presentation

**Figure 1.**
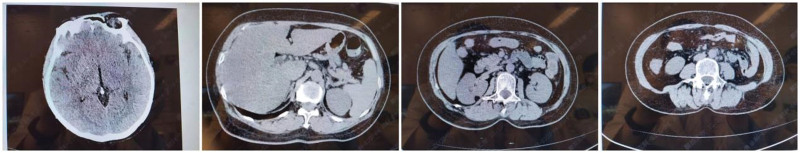
Computerized tomography (CT) of the patient’s head + whole abdomen on August 26, 2023.

Patient A, a 58-year-old Chinese woman, was admitted to the emergency department of our hospital on August 26, 2023, complaining of “fever with generalized muscle soreness for 2 days, abdominal pain and diarrhea for 1 day, and syncope for 1 time.” Two days before admission, the patient developed fever with muscle soreness and the highest body temperature was 40 °C. The patient visited the fever clinic of our hospital, and the nucleic acid test of 2019 novel coronavirus and influenza A and B virus antigen were negative. Blood routine (August 25, 2023): white blood cells 14.89 × 10^9^/L, neutrophils 13.22 × 10^9^/L, lymphocytes 1.41 × 10^9^/L, monocytes 0.22 × 10^9^/L, eosinophils 0.01 × 10^9^/L, basophils 0.01 × 10^9^/L, hemoglobin 143 g/L, platelets 284 × 10^9^/L. The high-sensitivity C-reactive protein (hs-CRP) was 190.6 mg/L. The outpatient doctor considered the diagnosis: fever to be diagnosed, and cefuroxime tablets 0.25 g orally Bid and ibuprofen oral suspension (2 g/100 mL) 10 mL orally 3 times a day were given. One day before admission, the patient developed abdominal pain, diarrhea, abdominal traction pain, 3 times of diarrhea, yellow loose stool, unknown volume, nausea, vomiting discomfort, 3 times of vomiting, no hematemesis, no hematochezia, no cough, no expectoration, no urgency, no urination pain, and came to the emergency department of our hospital for sudden syncope. The blood pressure of the patient was 74/45 mm Hg, and the patient was given fluid infusion to expand blood volume and anti-shock treatment immediately. Head computerized tomography (CT) showed no obvious abnormality. Abdominal CT showed thickening of the gastric antrum wall, multiple small lymph nodes in the abdominal cavity and retroperitoneum, blurred fat space with increased density, and a small amount of pelvic effusion (see Fig. 1). Blood routine (August 26, 2023): white blood cells 8.84 × 10^9^/L, neutrophils 7.44 × 10^9^/L, lymphocytes 1.09 × 10^9^/L, monocytes 0.30 × 10^9^/L, eosinophils 0.01 × 10^9^/L, basophils 0.00 × 10^9^/L, hemoglobin 151 g/L, platelets 198 × 10^9^/L. hs-CRP was 314.3 mg/L. Blood biochemistry (August 26, 2023): total bilirubin 23.3 µmol/L, alanine aminotransferase 83 U/L, aspartic transaminase 137 U/L, creatinine 419.2 µmol/L. Therefore, the patient was admitted to the emergency intensive care unit for monitoring and rescue treatment with abdominal pain, fever, and shock.

Considering that the patient had infection related symptoms such as fever, abdominal pain, and diarrhea, the serum procalcitonin level was 2.512 ng/mL on admission, but the urine routine, stool routine, and bacterial culture did not find the source of infection, and CT did not find clear infection lesions, so the patient’s past history was asked in detail. The patient denied a history of unclean diet, had not traveled recently, and had no known exposure to exogenous sources of infection. However, on February 31, 2023, the patient underwent skin biopsy in the dermatology clinic of our hospital. The pathological diagnosis was Behcet disease. Prednisone acetate tablets 40 mg oral quaque die (dose reduction 10 mg every 3 days until drug withdrawal) and thalidomide tablets 25 mg oral 3 times a day were given. On August 23, 2023, the patient was hospitalized in the Department of Orthopedics of our hospital due to severe osteoporosis and received an intravenous infusion of zoledronic acid injection 5 mg. She was discharged from the hospital on August 24, 2023, with fever and systemic muscle soreness on the day of discharge.

The patient was given intensive care immediately after admission, and massive fluid infusion to expand blood volume could not maintain normal blood pressure. Metaraminol was added to maintain circulatory stability, piperacillin–tazobactam was used to strengthen anti-infection, and glucocorticoids were used for anti-inflammatory treatment. Because the patient had infection related symptoms such as fever, abdominal pain, diarrhea and so on, the serum procalcitonin was 2.512 ng/mL after admission, and the Sepsis-related Organ Failure Assessment score^[3]^ was performed: respiratory system (0) + platelet count (0) + liver system (1) + circulatory system (2) + nervous system (1) + renal system (3) = 7. Therefore, the preliminary diagnosis was made: sepsis, septic shock, acute kidney injury, intestinal infection, and Behcet disease.

**Figure 2.**
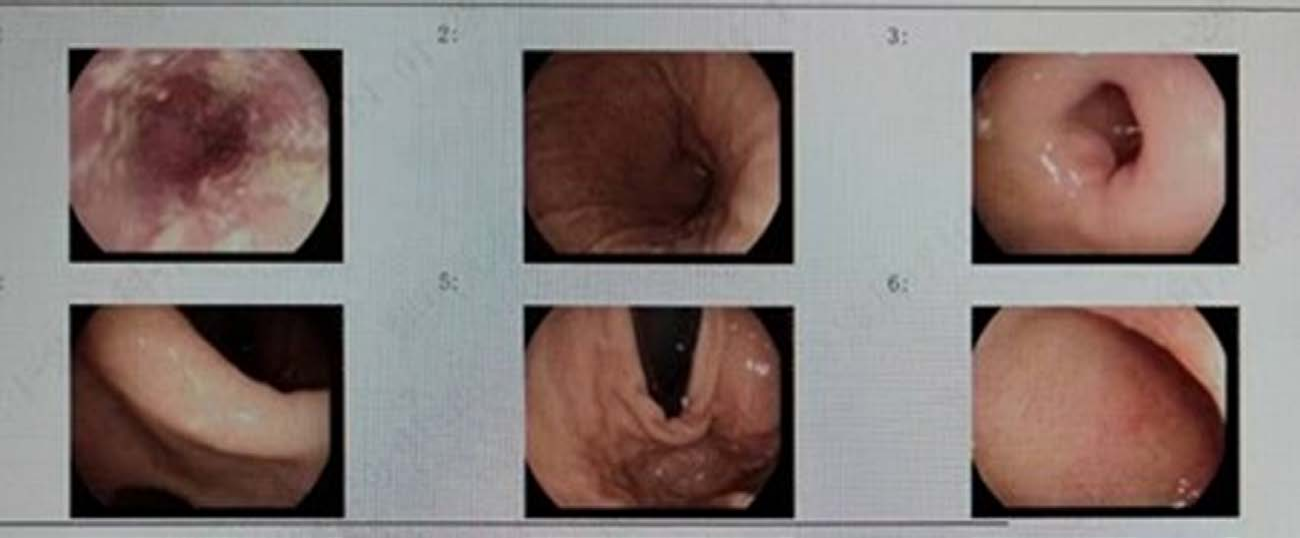
The patient underwent esophagoscopy + gastroduodenoscopy on August 26, 2023.

**Figure 3.**
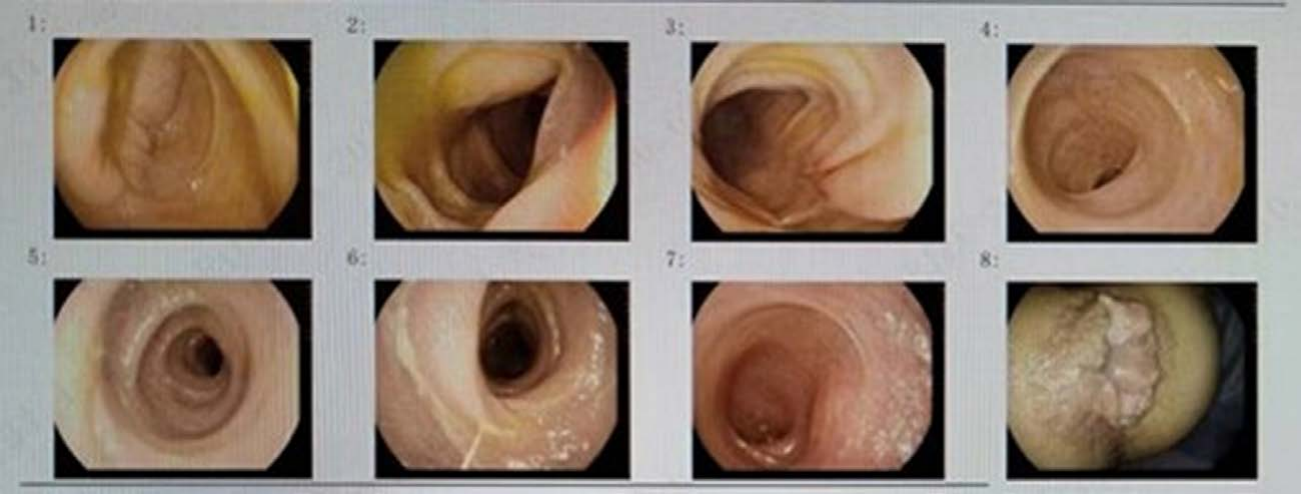
Patient colonoscopy + rectoscopy on August 26, 2023.

**Figure 4.**
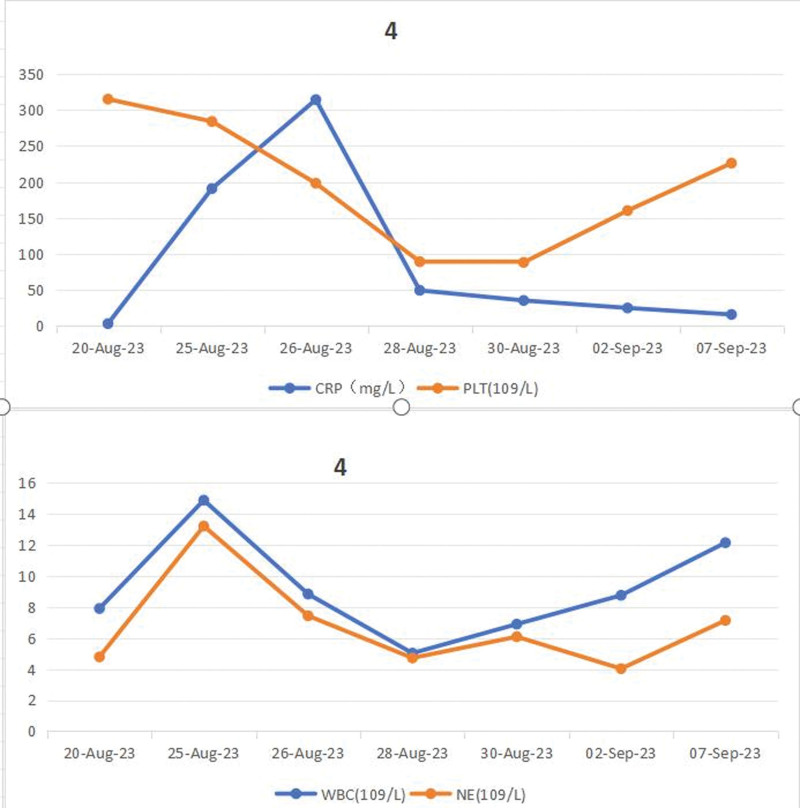
Changes in blood routine and high-sensitivity C-reactive protein during the course of the disease.

**Figure 5.**
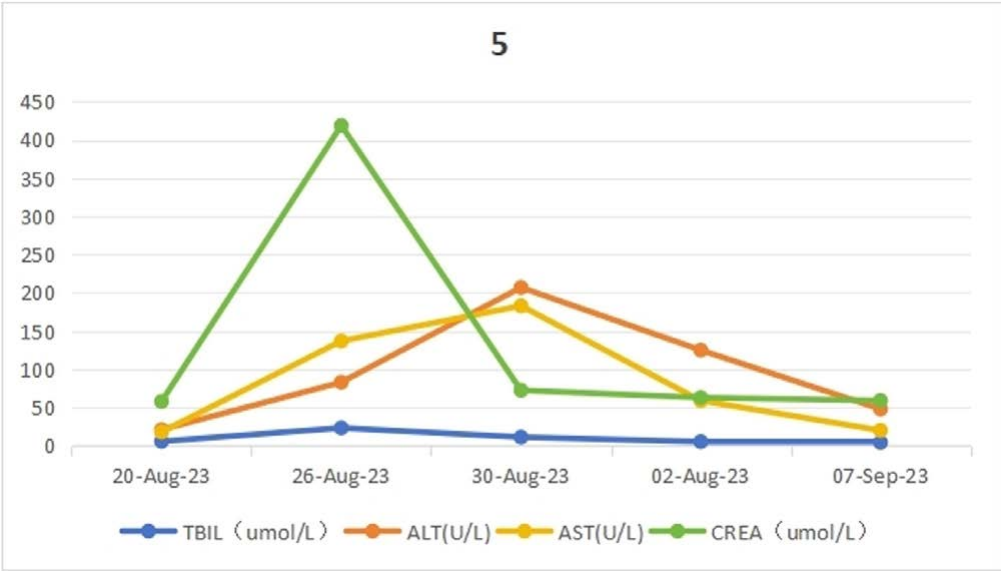
Changes in liver and renal function indices during the course of the disease.

**Figure 6.**
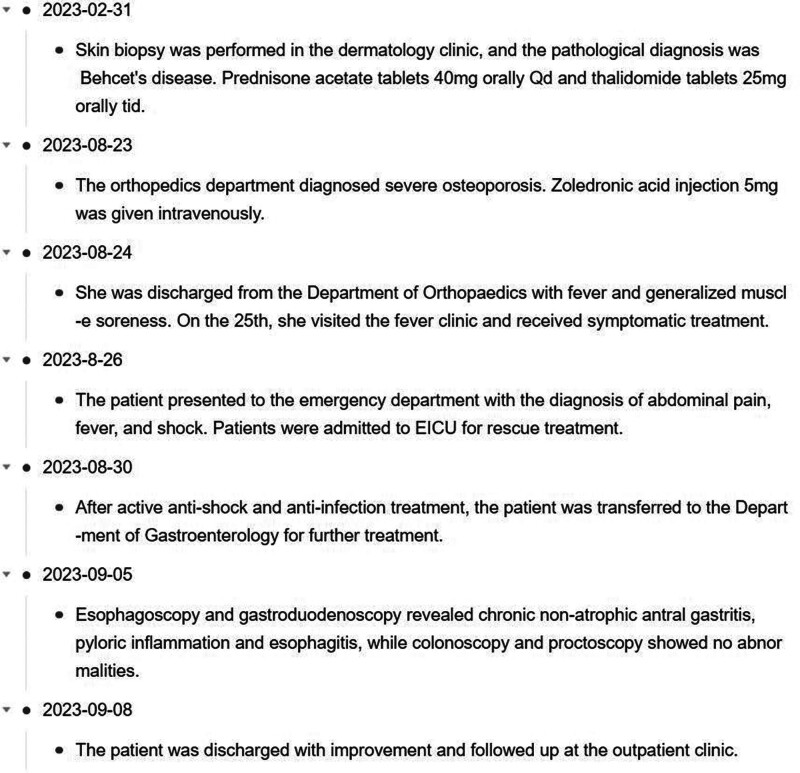
Patient clinical course and treatment timeline.

After active anti-shock and anti-infection treatment, the patient was transferred to the Department of Gastroenterology on August 30, 2023 after stable circulation. When the patient’s infection was under stable control, esophagoscopy and gastroduodenoscopy were completed on September 05, which revealed chronic non-atrophic antral gastritis, pyloric inflammation, and esophagitis (see Fig. 2), and colonoscopy and rectoscopy showed no abnormalities (see Fig. 3). On September 8, 2023, the patient was discharged with improvement and followed up in the outpatient clinic. The changes of blood routine and hs-CRP (see Fig. 4), liver and kidney function (see Fig. 5) during the course of the disease. A timeline of the patient’s clinical course and treatment (see Fig. 6).

## 3. Discussion

Sepsis is a life-threatening organ dysfunction caused by the imbalance of the body’s inflammatory response caused by infection.^[4,5]^ For patients with infection or suspected infection, sepsis can be diagnosed when the Sepsis-related Organ Failure Assessment score increases by ≥2 points from baseline.^[6]^ Sepsis is an important clinical problem faced by emergency and critical care medicine, with a mortality rate of more than 25%, while the mortality rate of patients with septic shock is more than 50%.^[7,8]^ Early identification and blocking the development of sepsis can significantly improve the prognosis of patients. In our case, the diagnosis of zoledronic acid-induced severe sepsis was established based on the temporal association between zoledronic acid exposure and the development of sepsis, clinical symptoms, and laboratory findings, in conjunction with consultation with a specialist clinician. In this case, the patient with severe sepsis induced by zoledronic acid recovered smoothly and was discharged from hospital through timely diagnosis and active rescue and treatment, and the occurrence of death adverse events was avoided.

Intravenous or high doses of oral nitrogenous bisphosphonates may cause acute phase reactions in up to 50% of patients receiving the first dose.^[1]^ The acute phase reaction is characterized by fever and muscle aches (an influenza-like symptom), which usually resolve within 48 hours and respond well to nonsteroidal anti-inflammatory drugs and antipyretic measures.^[1]^ Administration of acetaminophen 1 to 2 hours prior to treatment may reduce the likelihood of these reactions and may also be used to treat symptoms.^[1]^ The cause of the acute phase response is a transient increase in pyrogenic cytokines.^[2]^ In particular, γ/δ lymphocytes increased the production of interleukin-6 (IL-6) and tumor necrosis factor-α (TNF-α) after stimulation with dimethylallyl pyrophosphate.^[2]^ The present patient also had an acute phase reaction within 48 hours after the use of zoledronic acid injection, but this reaction is actually difficult to distinguish from the symptoms of acute infection. The only thing that can be done is to strengthen monitoring and observe the effect of symptomatic treatment measures.

Gastrointestinal adverse effects from bisphosphonates are common only with oral therapy. All levels of the GI tract are affected, from the lower esophagus to the colon. Although ulcers can occur in the esophagus, stomach, and duodenum, mucositis, flatulence, and diarrhea are more common.^[2]^ Because oral bisphosphonates are used much less frequently than parenteral formulations in cancer patients, most data on gastrointestinal toxicity of oral bisphosphonates come from studies of oral amino-bisphosphonates, ibandronate, alendronate, and risedronate in patients with osteoporosis.^[2]^ This patient also had severe gastrointestinal adverse reactions, including abdominal distension, abdominal pain, and diarrhea, and induced intestinal flora or toxin translocation, leading to enterogenous sepsis. The use of zoledronic acid caused mucosal inflammation in the gastrointestinal tract of the patient, leading to the damage of the gastrointestinal mucosal barrier. In addition, the patient had basic Behcet disease and long-term use of thalidomide and other immunosuppressive drugs, which eventually led to the displacement of intestinal flora or toxins and caused enterogenous sepsis. This is only a clinical etiology inference, and its mechanism needs to be verified by clinical experiments, but it also reminds us that when the patient has immunosuppression, the use of zoledronic acid needs to be cautious and strengthened monitoring.

Behcet disease is an autoinflammatory disorder characterized by recurrent painful mucocutaneous ulcers.^[9]^ The pathogenesis of Behcet disease remains to be elucidated, but it is generally described as a genetic disorder caused by an infectious source or trauma, resulting in immune activation, leading to inflammation and clinical symptoms.^[9]^ Immunoregulatory drugs such as colchicine, thalidomide, and corticosteroids are commonly used for treatment.^[9]^

We also observed that this patient had transient acute kidney injury which was considered to be caused by septic shock and the renal function returned to normal soon after the shock was corrected, which was different from the nephrotoxicity of zoledronic acid. Animal studies and clinical observations indicate that all bisphosphonates have the potential to cause acute tubular necrosis.^[2]^ The recovery time of acute kidney injury caused by renal tubular necrosis is longer than that of acute kidney injury caused by hypovolemia.

This study has some limitations. Firstly, zoledronic acid was not re-used to identify this adverse effect in our patient in depth. In addition, this is the first case of severe sepsis induced by zoledronic acid, and further observation and exploration of the mechanism of severe sepsis induced by zoledronic acid are needed in the future.

## 4. Conclusion

Severe sepsis induced by zoledronic acid should be considered a potentially serious adverse drug reaction, especially when the patient has immunosuppression, such as Behcet disease, whereas whether other bisphosphonate agents cause severe sepsis remains unknown. Clinicians, especially orthopedics, endocrinology and metabolism physicians, should be aware of this potential adverse effect. To ensure that zoledronic acid infusion is as safe as possible, patients are not allowed to leave medical monitoring for at least 24 to 48 hours after infusion, especially if they have immune-related conditions. When a patient has suspected sepsis after the use of zoledronic acid, the diagnosis should be confirmed and anti-infection related treatment should be carried out in time. In conclusion, severe sepsis induced by zoledronic acid is a rare but serious complication, and clinicians should be aware of this adverse event in time to avoid serious adverse consequences.

## Author contributions

**Conceptualization:** Lankai Liao, Ziyu Zhang.

**Data curation:** Lankai Liao, Ziyu Zhang.

**Formal analysis:** Lankai Liao.

**Investigation:** Lankai Liao.

**Methodology:** Lankai Liao.

**Resources:** Lankai Liao.

**Software:** Lankai Liao.

**Supervision:** Lankai Liao.

**Validation:** Lankai Liao

**Visualization:** Lankai Liao.

**Writing – original draft:** Lankai Liao, Ziyu Zhang.

**Writing – review & editing:** Lankai Liao.
